# ﻿*Doronicum micranthum* (Asteraceae, Senecioneae): a distinct new species from western Qinling, China

**DOI:** 10.3897/phytokeys.267.165470

**Published:** 2025-12-02

**Authors:** Zengfu Bai, Guiyuan Zhang, Zhihua Zhang, Anzhong Li, Hao Li, Geyang Wang, Xuelin Chen, Ji Zhang

**Affiliations:** 1 College of Life Sciences, Northwest Normal University, Lanzhou, 730070, Gansu, China; 2 Institute of New Rural Development, Northwest Normal University, Lanzhou, 730070, Gansu, China; 3 Gansu Xiaolongshan National Nature Reserve Management and Protection Center, Tianshui, 741020, Gansu, China

**Keywords:** Asteraceae, morphology, taxonomy, western Qinling

## Abstract

*Doronicum micranthum* (Asteraceae, Senecioneae), a new species from Hui County and Liangdang County in southeastern Gansu Province, China, is described and illustrated. This species is closely related to *D. stenoglossum* Maxim. and *D. conaense* Y.L.Chen, with which it shares the following characters: the ray florets are equal to or shorter than the involucre, and the small capitula are arranged in racemes at the stem apex. It differs by having swollen (vs. not swollen) rhizomes, smaller capitula (0.5–1.4 vs. 2–2.5 cm in diameter), and all florets’ achenes that are glabrous and lack a pappus (vs. achenes in all florets puberulent, pappus present, or glabrous and without pappus in ray florets, densely pubescent and bearing a pappus in disk florets).

## ﻿Introduction

*Doronicum* L. is primarily distributed in the temperate mountainous regions of Europe and Asia, as well as in northern Africa. Species of this genus grow in open or forested habitats from sea level up to 5000 m.

Álvarez Fernández (2003) recognized 26 species within *Doronicum*, while approximately 40 species have been reported worldwide in the *Flora of China*, with seven occurring in China (including four endemics) (Chen and Nordenstam 2011). All species are perennial herbs with alternate leaves; the basal leaves possess long petioles, whereas those of the cauline ones are sessile and semi-amplexicaul at the base; the capitula are radiate; and the achenes are cylindrical or obcylindrical with 10 longitudinal ribs. This genus is typically characterized by relatively large and brightly colored capitula. Some species are commonly cultivated as ornamentals, while others are valued for their medicinal properties (Chen 1999).

The genus is traditionally placed within the tribe Senecioneae (Cassini 1819; Bentham 1873; Hoffmann 1892; Nordenstam 1977; Bremer 1994; Fei et al. 2019), a placement also supported by chloroplast *ndhF* gene sequence data (Álvarez Fernández and Nieto Feliner 1999). However, the generic boundaries have undergone continuous changes, most notably through the exclusion of species now placed in *Arnica*, *Aster*, and *Nannoglottis* (Álvarez Fernández and Nieto Feliner 2000).

Located in the western Qinling Mountains’ biodiverse upper Jialing River basin, the Gansu Xiaolongshan National Nature Reserve spans Hui and Liangdang Counties in Longnan City, situated within a key ecological transition zone supported by complex geology and varied climates.

**Figure 1.**
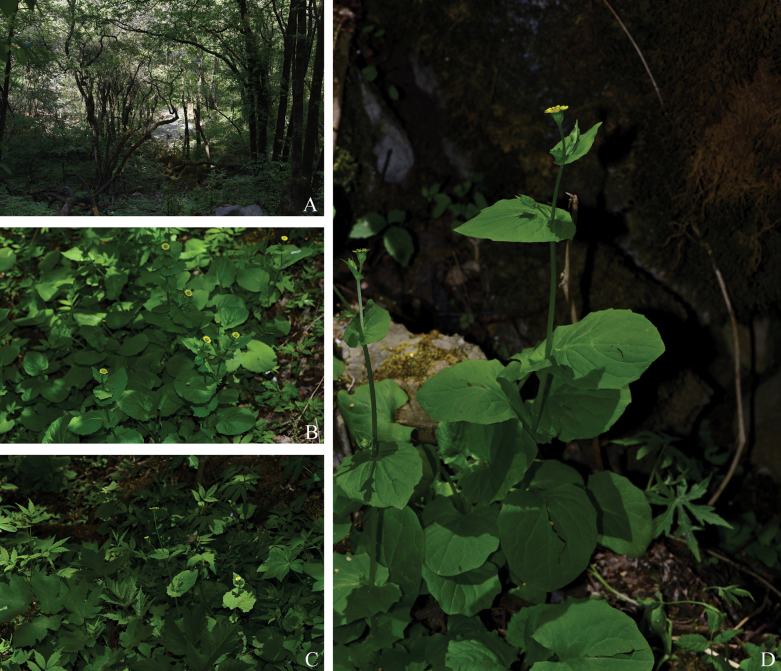
Habitat and habit of *Doronicum micranthum*.

**Figure 2.**
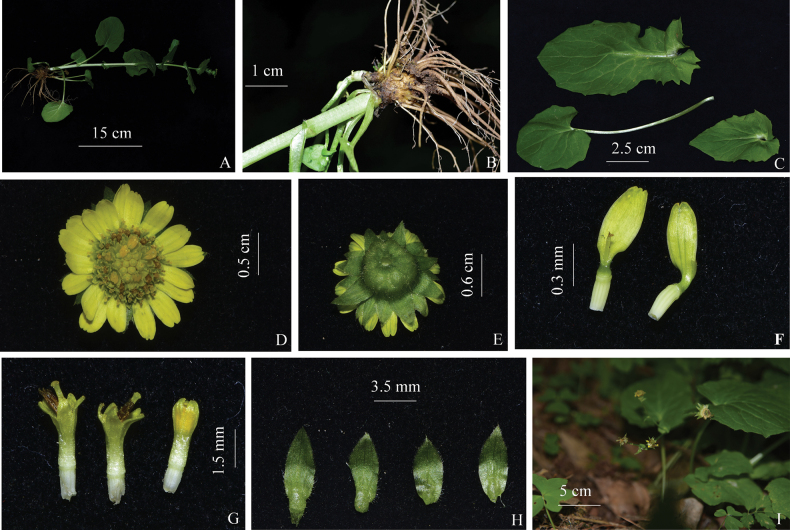
Detailed morphology of *Doronicum micranthum.***A.** Habit; **B.** Rhizome; **C.** Leaves; **D.** Top view of capitula; **E.** Bottom view of capitula; **F.** Ray florets; **G.** Disk florets; **H.** Phyllaries; **I.** Infructescence.

**Figure 3.**
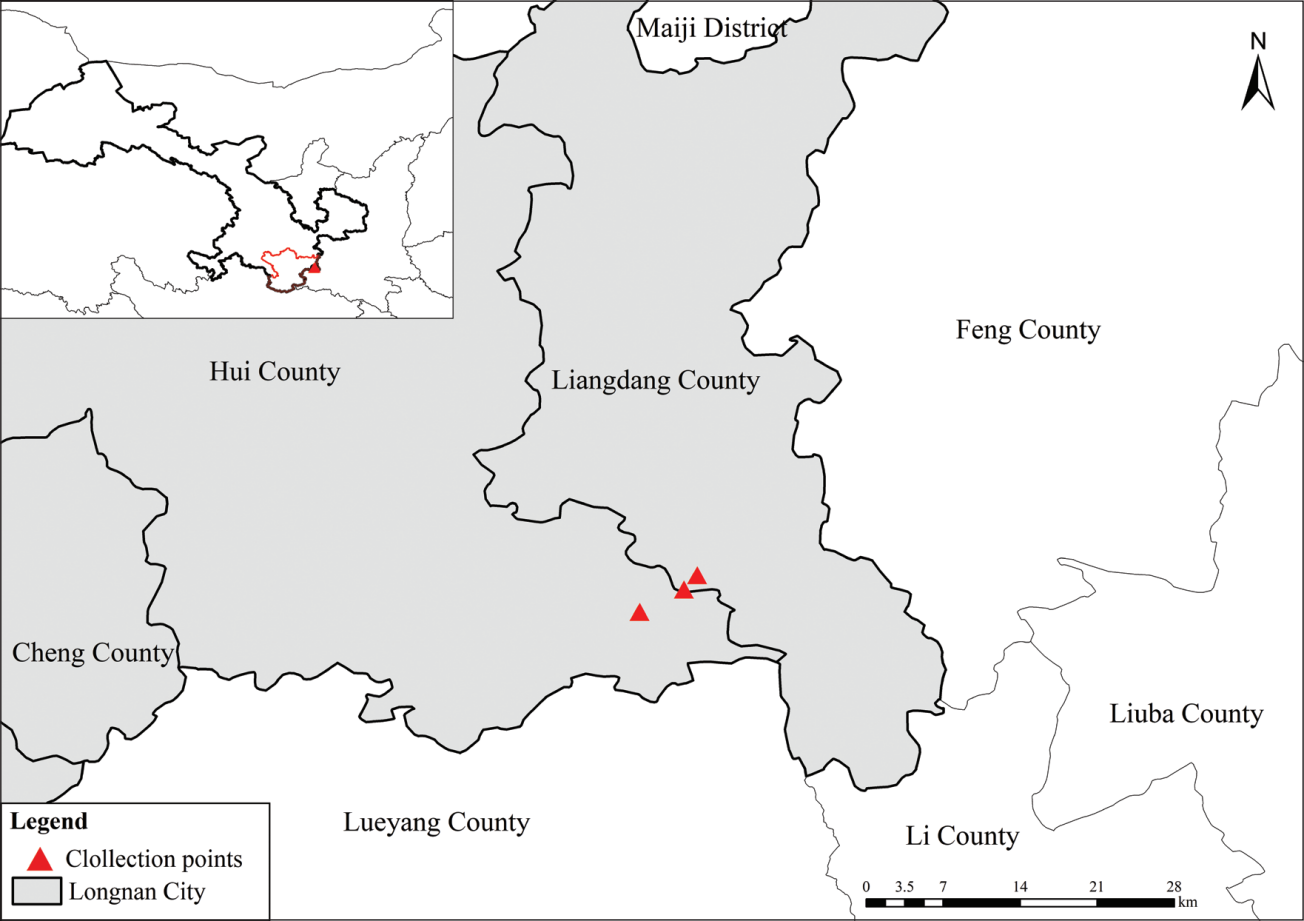
Distribution of *Doronicum micranthum*.

During the 2025 field investigation in Gansu Province, we discovered several populations of an unusual *Doronicum* species in Yuguan Town (Hui County) and Yunping Town (Liangdang County) (Fig. 3). The plants are morphologically distinct, characterized by very small capitula and completely glabrous achenes. Morphological comparisons with related taxa, literature review, and examination of herbarium specimens indicate its closest affinity with *D. conaense* and *D. stenoglossum*. However, it differs from these species in several aspects (Figs 1, 2). We therefore conclude that this plant represents a previously undescribed species, which we describe here as *Doronicum micranthum* Z.F.Bai & Xue L.Chen.

## ﻿Materials and methods

Measurements and descriptions of the new species are based on field-collected living material and the subsequent dried herbarium specimens. Voucher specimens were deposited in the Herbarium of the College of Life Sciences, Northwest Normal University (NWTC). Measurements were taken using a ruler, and micromorphological structures were observed and measured using a stereomicroscope. To assess the morphological differences among related species, we consulted relevant taxonomic literature and examined herbarium specimens from HNWP, KUN, NWTC, PE, and TIE (Chen 1998, 1999; Chen and Nordenstam 2011).

## ﻿Taxonomic treatment

### ﻿ Doronicum micranthum

Taxon classificationPlantaeAsteralesAsteraceae

Z.F.Bai & Xue L.Chen sp. nov.

58BC8FB0-78CE-54EE-8B9D-32CC0B3581A8

urn:lsid:ipni.org:names:77372725-1

Figs 1, 2

#### Type materials.

China • Gansu: Hui County, Yuguan town, in broad-leaved forests, alt. 2000 m, 10 May 2025, *Zengfu Bai BZF20250510005* (***holotype***NWTC!; ***isotypes***NWTC!).

***Paratypes*.** China • Gansu: Hui County, 10 May 2025, Z.F.Bai & Xue L.Chen 20250510006, 20250510007 (NWTC); 21 June 2025, Zhi Zheng 637 (NWTC).

#### Description.

Perennial herb. Rhizome fleshy, swollen, with numerous fibrous roots. Stems solitary, erect, 35–60 cm tall, unbranched or bearing only floral branches, green, sparsely glandular-puberulent. Basal leaves long-petiolate; leaf blades obovate-oblong, 1.2–7 cm long, 1–6.5 cm wide, apex rounded, base subcordate; petioles slender, 3.5–7.5 cm long, narrowly winged. Mid-cauline leaves ovate-oblong, sessile, 5.5–10.5 cm long, 3.5–7 cm wide, apex obtuse or mucronate, base cordate, semiamplexicaul, margin remotely dentate proximally, subentire distally, both surfaces subglabrous; upper cauline leaves ovate or ovate-lanceolate, 2–8 cm long, 1.5–6 cm wide, apex shortly acuminate, base cordate, semiamplexicaul. Capitula (including ray florets) 0.5–1.4 cm in diam., usually solitary; peduncles 2–13.5 cm long, glandular-pubescent. Involucres hemispheric, 4–8 mm long, 0.5–1.2 cm in diam.; phyllaries in 2–3 series, outer ones lanceolate, 1.5–2 mm wide, margin ciliate, abaxially glandular-pubescent on lower half, distally glabrous or subglabrous; inner ones narrowly lanceolate, ca. 1 mm wide, margin ciliate, abaxially glandular-pubescent; apex of all phyllaries long-acuminate, equal with or slightly longer than disk florets. Ray florets 18–25, yellow, 5–8 mm long; tube 1–2 mm long, glabrous; lamina oblong or oblong-elliptic, slightly spreading, 4–6 mm long, ca. 2 mm wide, 4-veined, apex 2–3-denticulate. Disk florets numerous; corolla yellowish green, 3–4 mm long; tube ca. 1 mm long; limb campanulate-funnelform, lobes ovate-triangular; anthers usually not exserted, ca. 1.5 mm long, base obtuse; style branches bifid, apex obtuse or truncate. Achenes brown, 10-ribbed. Pappus absent in all florets.

#### Phenology.

Flowering from May to June; fruiting from July to August.

#### Etymology.

The specific epithet refers to the smallest capitula among all known species in *Doronicum* in China. Hence, the Chinese name“小花多榔菊 (xiǎo huā duō láng jú)” is suggested.

#### Distribution and habitat.

*Doronicum micranthum* is hitherto known from Hui County (33°39'39.23"N, 106°19'45.32"E; 33°40'45.20"N, 106°21'56.13"E), Liangdang County (33°41'27"N, 106°19'35"E) of southeastern Gansu (Fig. 3). It grows in broad-leaved forests at elevations of 2000–2260 m.

#### Notes.

*Doronicum micranthum* is unambiguously distinguished from *D. conaense*, *D. stenoglossum*, and *D. calotum* by its characteristically small capitula (consistently less than 1.5 cm in diameter) and the complete absence of a pappus in all florets. Furthermore, *D. micranthum* possesses a prominently enlarged rhizome, a diagnostic feature not shared by the other three species discussed here.

In contrast, *D. conaense*, *D. stenoglossum*, and *D. calotum* all possess larger capitula (exceeding 1.5 cm in diameter) and a pappus, though its distribution varies: a pappus is present in all florets of both *D. stenoglossum* and *D. calotum*, whereas in *D. conaense*, the ray florets are epappose and the disk florets are pappose.

### ﻿Key to species of *Doronicum* in China

**Table:** 

1	Capitula less than 1.5 cm in diameter; pappus absent	***D. micranthum***
–	Capitula more than 1.5 cm in diameter; pappus present or absent	**2**
2	Ray florets as long as or shorter than involucre; capitula 1.5–2(–2.5) cm in diam., arranged in racemes	**3**
–	Ray florets distinctly longer than involucre; capitula 5–7 cm in diam., solitary, rarely 2	**4**
3	Peduncle thick, 3–8 cm, apex dilated; ray lamina oblong; achenes heteromorphic (ray florets glabrous, disk florets puberulent)	***D. conaense***
–	Peduncle slender, 1–1.5 cm; ray lamina linear; achenes homomorphic (all puberulent)	***D. stenoglossum***
4	Ray florets glabrous and epappose, disk florets pappose and hairy	**5**
–	All florets with pappus	**6**
5	Basal leaves elliptic; petiole 16–20 cm; ray floret tube glabrous	***D. oblongifolium***
–	Basal leaves obovate; petiole 4–15 cm, winged; ray floret tube glandular hairy	***D. turkestanicum***
6	Rhizomes slender, basal leaves obovate-spatulate or oblong-elliptic, involucre 3–3.5 cm in diam.; ray florets 2.2–2.8 cm	***D. calotum***
–	Rhizomes robust; basal leaves ovate or ovate-oblong, rarely orbicular, involucre 2–3 cm in diam.; ray florets 1.6–2.5 cm	***D. altaicum***

## Supplementary Material

XML Treatment for Doronicum micranthum

## ﻿Additional information

### Use of AI

No use of AI was reported.

### Data availability

All of the data that support the findings of this study are available in the main text.
